# Supplementary material for: Proof of Concept of the Contribution of the Interaction between Trait-like and State-like Effects in Identifying Individual-Specific Mechanisms of Action in Biological Psychiatry
Source: J Pers Med. 2022 Jul 23;12(8):1197. doi: 10.3390/jpm12081197 (PMC9332605; doi:10.3390/jpm12081197)
Supplement: Supplementary file 1 [file jpm-12-01197-s001.zip › jpm-1767952-supplementary.pdf]

METHODS

Participants

The study was conducted in the Adult and Late Life Depression Research Clinic and MRI Laboratory at the New York State Psychiatric Institute (NYSPI). All procedures were approved by the NYSPI Institutional Review Board. Eligible participants were men and women aged 24-65 years, who met Diagnostic and Statistical Manual IV (DSM-IV) (American Psychiatric Association 2000) criteria for non-psychotic MDD, had a 24-item HRSD score ≥ 16, were right-handed, had no contraindications to MRI, gave informed consent, and complied with study procedures.

Study design

Study procedures are described in a previous report of clinical findings (Rutherford et al., 2017). Briefly, 50 patients were enrolled in an 8-week antidepressant clinical trial, randomizing participants to Placebo-controlled and Open groups. At baseline, patients underwent initial evaluation, eligibility was assessed, and pre-randomization HRSD scores and outcome expectancy (operationalized as their belief regarding the probability of receiving medication: 0 vs. 25% vs. 50% vs. 100%) were measured. fMRI scan 1 was performed as soon as possible after this visit, within 1 week. Following fMRI scan 1, patients' level of outcome expectancy was manipulated by randomization to either the Placebo-controlled group (50% chance of receiving active treatment) or the Open group (100% chance of receiving active treatment), and patients were informed of the results of randomization (which was the means of manipulating outcome expectancy). Outcome assessors were blinded to group assignment. At the Week 0 visit, post-randomization outcome expectancy and depression scores were measured, with participants having this additional information. Participants in the Placebo-controlled group were blinded to treatment assignment within the

group. fMRI scan 2 was then performed within 1 week of the Week 0 visit, after which either citalopram or a placebo pill was administered. Thus, both pre- and post-randomization outcome expectancy measurements and fMRI scans 1-2 were obtained before patients received any medication. HRSD was measured weekly over the 8-week clinical trial.

Materials

Masked Emotional Face task

In this task, participants viewed black and white pictures of human faces displaying fearful, sad, happy, or neutral emotional expressions taken from a standardized serie (Ekman, 1976). Stimuli were masked so that an emotional face was presented for 33ms followed by 160ms presentation of a neutral face. Pilot testing and post-scan debriefing indicated that participants are only consciously aware of observing one face per trial. Following the face presentations, participants obtain affective ratings using a grid displaying the dimensions of valence (pleasant-unpleasant) and arousal (excited-sleepy) as visual analogue scales on the x- and y-axes, respectively, ranging from 1 to 100 in each dimension. Patients viewed 1 run of 120 trials comprising 30 presentations of each emotional valence (sad, happy, fearful, and neutral) followed by the neutral face. Each run scanned approximately 450 functional images (TR=2000ms).

Monetary incentive delay task

In this task, participants viewed one of three cue shapes, fixate on a crosshair as they wait a variable interval, respond to a target appearing for a variable amount of time with a button press, then view a feedback screen. Cues signal potential gain outcomes (open circles), potential loss outcomes (open squares), or no monetary outcomes (open triangles). One to three horizontal lines within the gain and loss cues signal the possibility of winning $0.20,

$1.00, or $5.00 or losing $0.20, $1.00, or $5.00. Depending on the incentive cue, subjects win or lose money based on whether they press a button during target presentation. Feedback screens notify participants of the trial's result and their cumulative money total. Participants' likelihood of successful button pressing is manipulated via an adaptive timing algorithm that follows the subject's performance on the preceding 6 trials and alters target duration such that subjects will succeed on approximately 66% of trials. Participants complete 2 runs of 72 trials lasting 6 s each.

Image acquisition

Images were obtained on a GE Signa 3-T whole body scanner (Milwaukee, WI) operating the E2-M4 platform using a quadrature head coil in receive mode. T1-weighed sagittal localizing images were used to position axial functional images parallel to the anterior-posterior commissure (AC-PC) line. A 3D spoiled gradient recall (SPGR) image was acquired for coregistration with axial echoplanar images and a reference brain from the Montreal Neurological Institute (MNI). Axial echoplanar images (TR = 2000 ms, TE = 28 ms, 77° flip angle, single excitation per image, slice thickness 3.54 mm, 1.0 mm gap, 24 cm × 24 cm field of view, 64 × 64 matrix) were obtained to provide an effective resolution of 3.75 mm × 3.75 mm × 3.5 mm and whole brain coverage, with 35 slices in each imaging volume and 452 volumes per run.

Image pre-processing

SPM8 (http://www.fil.ion.ucl.ac.uk/spm/) under MATLAB 2014B was used to preprocess the functional imaging data. The preprocessing procedure included the following steps: (a) slice-timing correction using the middle slice of each run as the reference image; (b) motion correction for three translational directions and rotations using a rigid-body

transform; (c) spatial normalization to the standard MNI template using a hybrid algorithm of affine transform and nonlinear warping. Each participant's high-resolution structural image (fSPGR) was normalized to the template, and these subject-specific warping parameters were then used to normalize the functional images to the same template; (d) reformatting of the normalized functional images to 3x3x3 mm voxels; (e) Gaussian spatial filtering with a FWHM of 8 mm. A discrete cosine transform-based high-pass filter with a basis function length of 128s was also used to remove low-frequency noise, such as scanner drift, from the baseline image intensity.

Functional Image Analyses

Using SPM8, we performed an individual-level analysis (first-level) to detect task-related (face stimulation-related) activity within each participant. We then performed group-level analysis (second-level) to detect random effects of task-related activity. We conducted the first-level analysis using the general linear model (GLM), as implemented in SPM8, to model the data for each participant, with 4 independent functions and a constant for each run. The first 2 independent functions corresponded to 2 events recorded in the task, each generated by convolving a canonical hemodynamic response function (HRF) with a boxcar function (BCF) derived from the onsets and durations of each event, facial presentation, and participant rating. The second 2 independent functions were generated by a separate amplitude modulation of the facial stimulation function with each rating score, arousal score, and valence score. The model was estimated using the Restricted Maximum Likelihood (ReML) algorithm. Task-related T contrast images were generated using SPM8.

We implemented a Bayesian posterior inference approach (Surguladze et al., 2005) for the second-level analysis of the contrast images generated from the first-level GLM-based analysis to detect the random effects of task-related activity within and between the groups.

We used a posterior probability of 97.50% as the threshold of significant posterior probability maps (PPMs), a rigorous threshold in Bayesian inference, to ensure that reported findings are true positives (Friston and Penny, 2003). We extracted ROI BOLD data based on the PPM images within those regions, showing significant group effects (open vs. PC group) in the differences between scan 2 and scan 1 on the contrast images of sad vs. neutral faces. The amygdala ROI was defined based on a brain atlas (Amunts et al., 2005), and the signal was extracted from the ROI by averaging BOLD signals across all voxels within the ROI for each contrast, for each patient. We entered the ROI data into further mediation analyses.
